# Reduced Binding between Omicron B.1.1.529 and the Human ACE2 Receptor in a Surrogate Virus Neutralization Test for SARS-CoV-2

**DOI:** 10.3390/v15061280

**Published:** 2023-05-30

**Authors:** Tove Hoffman, Linda Kolstad, Dario Akaberi, Josef D. Järhult, Bengt Rönnberg, Åke Lundkvist

**Affiliations:** 1 Department of Medical Biochemistry and Microbiology, Zoonosis Science Center (ZSC), Uppsala University, 752 37 Uppsala, Sweden; linda.kolstad@imbim.uu.se (L.K.); dario.akaberi@imbim.uu.se (D.A.); bengt.ronnberg@gmail.com (B.R.); ake.lundkvist@imbim.uu.se (Å.L.); 2 Department of Medical Sciences, Zoonosis Science Center (ZSC), Uppsala University, 751 85 Uppsala, Sweden; josef.jarhult@medsci.uu.se

**Keywords:** SARS-CoV-2, Omicron, antibody-mediated blockage, ACE2, spike, receptor binding domain

## Abstract

The current gold standard assay for detecting neutralizing antibodies (NAbs) against severe acute respiratory syndrome coronavirus 2 (SARS-CoV-2) is the conventional virus neutralization test (cVNT), which requires infectious virus and a biosafety level 3 laboratory. Here, we report the development of a SARS-CoV-2 surrogate virus neutralization test (sVNT) that, with Luminex technology, detects NAbs. The assay was designed to mimic the virus–host interaction and is based on antibody blockage between the human angiotensin-converting enzyme 2 (hACE2) receptor and the spike (S) protein of the Wuhan, Delta, and Omicron (B.1.1.529) variants of SARS-CoV-2. The sVNT proved to have a 100% correlation with a SARS-CoV-2 cVNT regarding qualitative results. Binding between the hACE2 receptor and the S1 domain of the B.1.1.529 lineage of the Omicron variant was not observed in the assay but between the receptor and an S1 + S2 trimer and the receptor binding domain (RBD) in a reduced manner, suggesting less efficient receptor binding for the B.1.1.529 Omicron variant. The results indicate that the SARS-CoV-2 sVNT is a suitable tool for both the research community and the public health service, as it may serve as an efficient diagnostic alternative to the cVNT.

## 1. Introduction

In December 2019, the coronavirus disease 2019 (COVID-19) outbreak was recognized in Wuhan, China [1]. Since then, the disease has spread globally and been classified as a pandemic—with more than 686 million confirmed infections and more than 6.8 million deaths globally as of 24 April 2023 [2]. The causative agent is severe acute respiratory syndrome coronavirus 2 (SARS-CoV-2), which belongs to the Betacoronavirus genus of the *Coronaviridae* family [3]. It is an enveloped, single-stranded RNA virus composed of 16 non-structural proteins and four structural proteins; the envelope (E), membrane (M), nucleocapsid (N), and spike (S) proteins [3]. The surface-anchored S protein comprises two domains/subunits: S1 and S2. The S1 domain contains the receptor binding domain (RBD; aa_wild type_: 319-541) that specifically recognizes the human angiotensin-converting enzyme 2 (hACE2)—the main functional receptor for viral attachment and entry in humans and the primary target for neutralizing antibodies (NAbs)—while the S2 domain is responsible for membrane fusion [3]. On virions, the S protein is present in a trimeric form with three S1 heads on a trimeric S2 stalk [4]. NAbs against the virus can block viral entry and are needed to reduce the spread of the virus.

Vaccines against SARS-CoV-2 were quickly developed as a joint effort by the research community and the vaccine industry. Several vaccines were approved and out in the distribution chains in less than one year. The mRNA vaccines Spikevax (mRNA-1273) and Comirnaty (BNT162b2) manufactured by Moderna and Pfizer/BioNTech, respectively, have been widely distributed and encode a stabilized full-length ectodomain version of the S protein of the wild type Wuhan-Hu-1 variant of SARS-CoV-2 [5]. As the COVID-19 pandemic has progressed, the vaccination rates have increased globally since the protective immunity in vaccinees has assisted in the relaxation of containment measures globally and in a decreased disease severity. However, the effort of preventing the spread of SARS-CoV-2 is continuously hampered by a short-lived immunity and the emergence of new variants of the virus due to mutations, such as the Alpha (B.1.1.7), Beta (B.1.351), Delta (B.1.617.2) and Omicron (B.1.1.529) variants [6]. In November 2021, the World Health Organization (WHO) declared Omicron a variant of concern [7]. Since then, the variant has become the dominating circulating variant and evolved into several lineages/subvariants, such as B.1.1.529, BA.1, BA.2, BA.3, BA.4, and BA.5 [6]. The Omicron variant has a high number of mutations, especially in the S protein, compared to earlier variants. Fifteen of the mutations are located in the RBD of the protein [8], and the mutations are likely to be associated with the observed immune evasion (escape of infection- and vaccine-induced immunity) and increased transmissibility of Omicron. Therefore, increased knowledge about receptor binding and immune evasion mutations is important to limit the spread of SARS-CoV-2.

The conventional virus neutralization test (cVNT) is the gold standard for detecting NAbs against SARS-CoV-2. However, the method requires infectious virions and, therefore, must be performed in a biosafety level 3 (BSL-3) facility. Furthermore, the method is time-consuming and requires trained personnel. Serological assays able to efficiently detect NAbs against SARS-CoV-2 are therefore needed. In this study, we have developed a surrogate virus neutralization test (sVNT) that, with Luminex technology, detects blocking antibodies targeting the S protein of SARS-CoV-2 in a species- and isotype-independent manner and present data that indicate that the method can successfully be used as a substitute for the cVNT. Furthermore, we report a reduced binding between the B.1.1.529 lineage of the Omicron variant of SARS-CoV-2 and the hACE2 receptor.

## 2. Materials and Methods

### 2.1. Serum Samples

The human control panel consisted of: (i) Sera from Swedish confirmed COVID-19 patients (*n* = 54) infected with the Wuhan variant, (ii) Pooled sera collected from individuals (*n* = 8) previously infected with the Wuhan variant, (iii) Serum collected from individual (*n* = 1) previously infected with the Omicron variant, (iv) WHO’s international standard for anti-SARS-CoV-2 (NIBSC code: 20/136), (v) WHO’s international reference panel for anti-SARS-CoV-2 (NIBSC code: 20/268 (includes samples: 20/140 (Lowest antibody (Ab) levels), 20/142 (Negative human sera), 20/144 (Low Ab levels), 20/148 (Mid Ab levels), and 20/150 (High Ab levels)), (vi) The European Centre for Disease Prevention and Control’s (ECDC) SARS-CoV-2 serological external quality assessment panel (EQA A-F) (includes also sera known to be positive for the presence of antibodies against the human coronaviruses 229E, HKU1, OC43, and NL63 as well as acute Epstein-Barr virus/Cytomegalovirus sera) [9], (vii) ECDC’s SARS-CoV-2 serological standards (JCR 017 and JCR 018) [9], (viii) Pre-COVID-19 negative control sera (*n* = 200) collected in 2018 from randomly selected blood donors, (ix) WHO’s international standard for Middle East respiratory syndrome coronavirus (MERS-CoV) immunoglobulin G (human) (NIBSC code: 19/178), (x) Rheumatoid arthritis serum (human) (NIBSC code: 64/002), (xi) Standard for Varicella-Zoster virus (VZV) antibodies (human) (NIBSC code: 90/690), (xii) Sera from individuals previously infected with *Borrelia* (*n* = 3). See Hoffman et al. [10] for information about the number of days between symptom onset/PCR-positivity and serum sampling for the confirmed COVID-19 patients and the convalescents. The serum panels were stored at +4 °C or −20 °C.

### 2.2. SARS-CoV-2 sVNT

**Figure 1:**
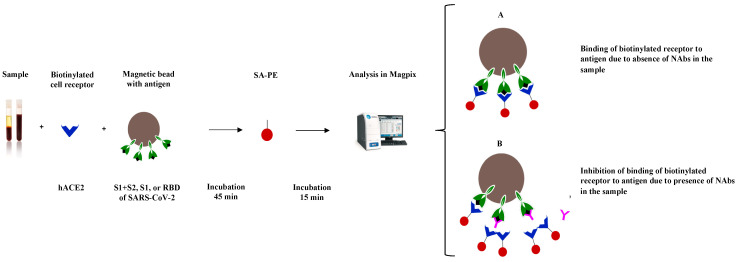
Principle of the SARS-CoV-2 surrogate virus neutralization test, where neutralizing antibodies block the binding of hACE2 to conjugated SARS-CoV-2 S proteins. S—spike; S1—the domain of the spike protein involved in receptor binding; S2—membrane fusion domain of the spike protein; RBD—receptor binding domain; hACE2—human angiotensin-converting enzyme 2; SA-PE—streptavidin-phycoerythrin; NAb—neutralizing/blocking antibody.

The principle of the SARS-CoV-2 sVNT is presented in Figure 1.

#### 2.2.1. Conjugation

In brief, 10 µg of recombinant SARS-CoV-2 S1 proteins (Table 1) were coupled to 2.5 × 10^6^ carboxylated differentially color-marked magnetic beads (MagPlex microspheres, Luminex Corp., Austin, TX, USA) using sulfo-N-hydroxysulfosuccinimide (50 mg/mL, in H_2_O) (24510, Pierce Biotechnology, ThermoFisher, Waltham, MA, USA) and 1-ethyl-3-[3 dimethylaminopropyl]carbodiimide hydrochloride (50 mg/mL, in H_2_O) (03449-1G, Sigma Aldrich, Merck, Darmstedt, Germany) according to the manufacturer’s instructions. Conjugated beads were stored in StabilGuard™ Immunoassay Stabilizer (SG01-0050, DIARECT, BBI Solutions, Crumlin, UK) at −20 °C. The coupling efficiency was evaluated using positive control sera.

#### 2.2.2. Binding Inhibition

**Figure 2:**
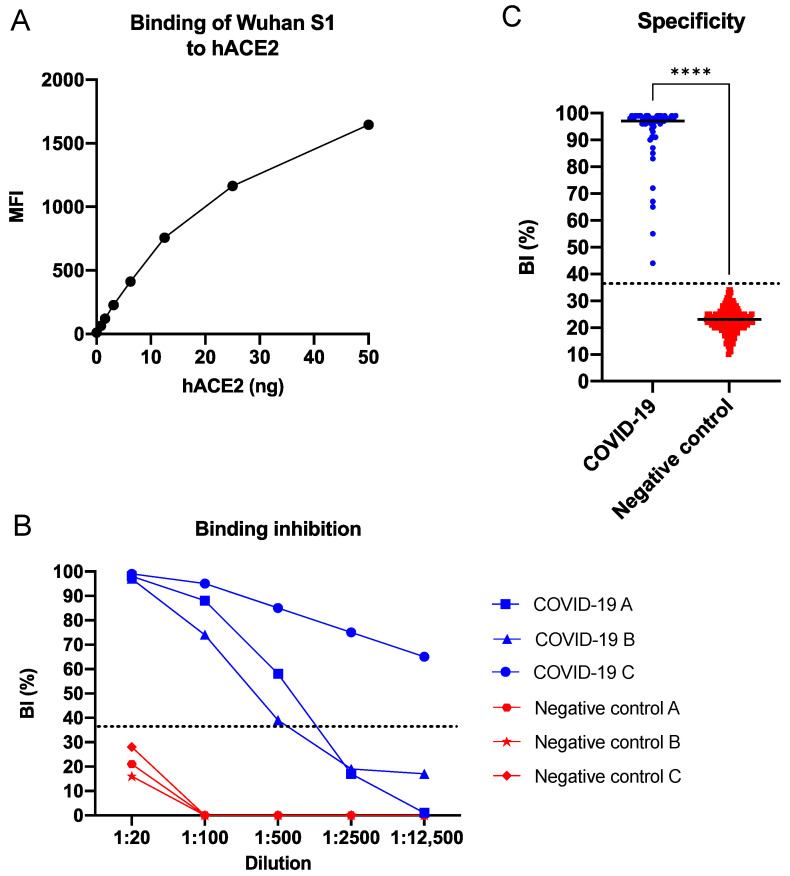
Wuhan S1 SARS-CoV-2 sVNT. (**A**) Binding of biotinylated hACE2 to conjugated SARS-CoV-2 Wuhan S1 protein. (**B**) Inhibition of hACE2—SARS-CoV-2 S1 interaction by a selected number of sera from patients with COVID-19. (**C**) Specificity of the SARS-CoV-2 sVNT in singleplex format. The COVID-19 group (in blue) consisted of sera from patients (*n* = 54), and the negative control group (in red) consisted of pre-COVID-19 blood donor sera (*n* = 200). Data were analyzed using the Mann–Whitney test, and a *p*-value < 0.05 was considered significant. **** = *p*-value < 0.0001. sVNT—surrogate virus neutralization test; BI—binding inhibition; S1—domain of the spike protein involved in receptor binding; hACE2—human angiotensin-converting enzyme 2; dotted line indicates a cut-off of 36%; black horizontal lines indicate median.

For the determination of binding inhibition (BI), a 5 μL serum sample in 20 μL phosphate-buffered saline (PBS) with tris(hydroxymethyl)aminomethane (TRIS) and Tween (PBSTT) and 25 μL of biotinylated hACE2 (20 ng (Figure 2A); 10108-H02H-B, SinoBiological, Beijing, China) were incubated for 45 min with 50 μL vortexed and sonicated coupled beads (25 beads/μL PBSTT), giving a final serum dilution of 1:20. Subsequently, the suspension was washed with 100 μL PBS, followed by the addition of 100 μL streptavidin-phycoerythrin (phycoerythrin served as the signal molecule) (2 μg/mL, in PBSTT) (SA10044, Invitrogen, ThermoFisher Scientific, Waltham, MA, USA), a 15 min incubation, another PBS wash, addition of 100 μL PBS, and briefly mixing the final reactions on a plate shaker. All incubations were performed in the dark, at room temperature, and on a plate shaker at 400 rpm. The median fluorescence intensity (MFI) was determined using a reaction volume of 50 μL in the MagPix instrument (Luminex Corp.). The BI for each sample was calculated using Equation (1).

(1) $$BI\left(\%\right)=\left(1-\left(\frac{MFI\;sample}{MFI\;PBSTT}\right)\right)\times 100$$

Neutralization/blocking titers were determined by a five-fold serial dilution in five steps, starting at 1:20.

#### 2.2.3. Assay Cut-Off

The assay cut-off for positivity was calculated as the average BI % plus three standard deviations of SARS-CoV-2 antibody-negative human control sera (*n =* 200).

### 2.3. SARS-CoV-2 cVNT

#### 2.3.1. Cells and Virus

Vero E6 cells were cultured in Dulbecco’s Modified Eagle Medium (DMEM) (41966029, Gibco, Thermo Fisher Scientific, Waltham, MA, USA) supplemented with 10% Fetal Bovine Serum (FBS) (10500064, Gibco, Thermo Fisher Scientific, Waltham, MA, USA) and 1X penicillin-streptomycin (PA333, Sigma-Aldrich, Merck, Darmstedt, Germany) and incubated at 37 °C, 5% CO_2_. The SARS-CoV-2 used in this study is a Swedish isolate from 2020 [11].

#### 2.3.2. Plaque Reduction Neutralization Test

Vero E6 cells were seeded at a density of 2 × 10^5^ cells/well in 24 wells plates and incubated overnight. On the day of the assay, serum samples were heat inactivated by incubation at 56 °C for 30 min and diluted five (1:5) and ten times (1:10) in DMEM (2% FBS, 1X penicillin-streptomycin). The serum samples were then diluted twice more by mixing the same volumes of the serum dilutions with the virus, giving a final 1:10 and 1:20 dilution of the serum samples and a virus dilution giving approximately 50 plaques forming units (PFU)/0.1 mL. The serum-virus mixtures were then incubated at 37 °C with 5% CO_2_ for 1 h, and 100 µL was used to inoculate Vero E6 cells. Infection-controls (non-neutralized virus) were inoculated with 100 µL of a 1:1 mix of virus dilution (~50 PFU) and DMEM (2% FBS, 1X penicillin-streptomycin) that was also previously incubated for 1 h at 37 °C with 5% CO_2_. Uninfected-cell controls were mock-inoculated with 100 µL of DMEM (2% FBS, 1X penicillin-streptomycin).

The inoculated cells were incubated at 37 °C with 5% CO_2_ for 1 h, and plates were gently shaken every 10 min to avoid drying the cell monolayer. Following 1 h incubation, cells were overlayed with 500 µL of a 1:1 mix of 1.6% noble agar solution in water (A5431, Sigma-Aldrich, Merck, Darmstedt, Germany) and 2X MEM (11935046, Gibco, Thermo Fisher Scientific, Waltham, MA, USA) supplemented with 2% FBS and 1X penicillin-streptomycin. Plates were incubated for 72 h, and cells were stained with 500 µL of a 3% neutral red solution obtained by diluting 1.5 mL of 0.33% neutral red solution (N2889, Sigma-Aldrich, Merck, Darmstedt, Germany) into a final volume of 50 mL of PBS. After 4 h incubation, the neutral red solution was removed, plaques were counted, and the percentage of the inhibition of plaque formation was calculated using Equation (2).

(2) $$Inhibition\left(\%\right)=\left(\frac{Average\;n\;plaques\;in\;infection\;control\;wells-Average\;n\;plaques\;in\;test\;wells}{Average\;n\;plaques\;in\;infection\;control\;wells}\right)\times 100$$

Sera that inhibited plaque formation by 50% or more at 1:20 dilution were considered positive for previous SARS-CoV-2 infection.

### 2.4. Statistical Analyses and Data Visualization

Statistical analyses and data visualization were performed using GraphPad Prism version 9.0.1 (San Diego, CA, USA). The data were analyzed using the Mann–Whitney test, and a *p*-value < 0.05 was considered significant.

## 3. Results

### 3.1. SARS-CoV-2 sVNT in Singleplex Format

#### 3.1.1. Cut-Off Determination

The positivity cut-off value for the BI % in human sera, using the antigen Wuhan S1 and 200 negative control sera, was determined as 36%. All of the analysed positive control sera (*n* = 54) exhibited a BI above the cut-off (Range: 44–99%; Median: 97%), and all of the analysed negative control sera (*n* = 200) exhibited a BI below the cut-off (Range: 10–34%; Median: 23%) (Figure 2C).

#### 3.1.2. Binding Evaluation

The binding between the SARS-CoV-2 Wuhan S1, conjugated to magnetic beads, and biotinylated hACE2 was evaluated. The binding between the receptor and the antigen was found to be dose-dependent (Figure 2A), to be blocked by the COVID-19 sera (Figure 2B), and to be specific (Mann–Whitney test, *p*-value < 0.0001) (Figure 2C). The sensitivity (probability of a positive test result among those with the target condition) and the specificity (probability of a negative test result among those without the target condition) of the sVNT based on the Wuhan S1 antigen were both determined to be 100%. The positive predictive value (PPV; probability of having the target condition given a positive test result) and the negative predictive value (NPV; probability of not having the target condition given a negative test result) were also both determined as 100%.

#### 3.1.3. Inter and Intra Assay Variability

The coefficient of variability (CV) of the singleplex format of the SARS-CoV-2 sVNT, i.e., the measures of repeatability (intra) and reproducibility (inter) of the assay, was determined to be 10% and 8% for the CV_Intra_ and CV_Inter_, respectively.

### 3.2. SARS-CoV-2 sVNT in Multiplex Format

**Figure 3:**
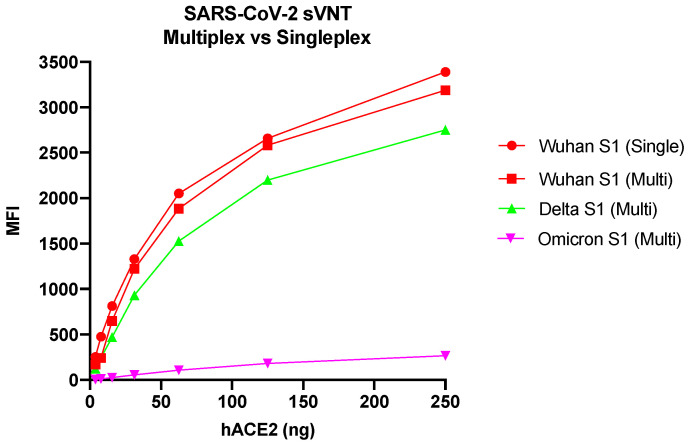
Evaluation of the SARS-CoV-2 sVNT in single versus multiplex format. sVNT—surrogate virus neutralization test; hACE2—human angiotensin-converting enzyme 2; MFI—median fluorescence intensity; S1—domain of the spike protein involved in receptor binding; Wuhan, Delta, Omicron (B.1.1.529)—variants of SARS-CoV-2.

The multiplex format of the SARS-CoV-2 sVNT was initially evaluated using Wuhan S1, Delta S1, and B.1.1.529 Omicron S1 antigens from SinoBiological (Table 1). Due to the absence of binding between the hACE2 receptor and the B.1.1.529 Omicron S1 antigen, the amount of the hACE2 receptor was increased up to 250 ng. The highest amount of the hACE2 receptor (250 ng) generated the highest MFI both in the singleplex format of the assay when only the Wuhan S1 antigen was used and in the multiplex format when all three antigens were used (Figure 3). At lower concentrations of the ACE2 receptor, a decreased signal was observed. Binding could not be detected between the ACE2 receptor and the Omicron S1 antigen despite increasing the amount of receptor.

**Figure 4:**
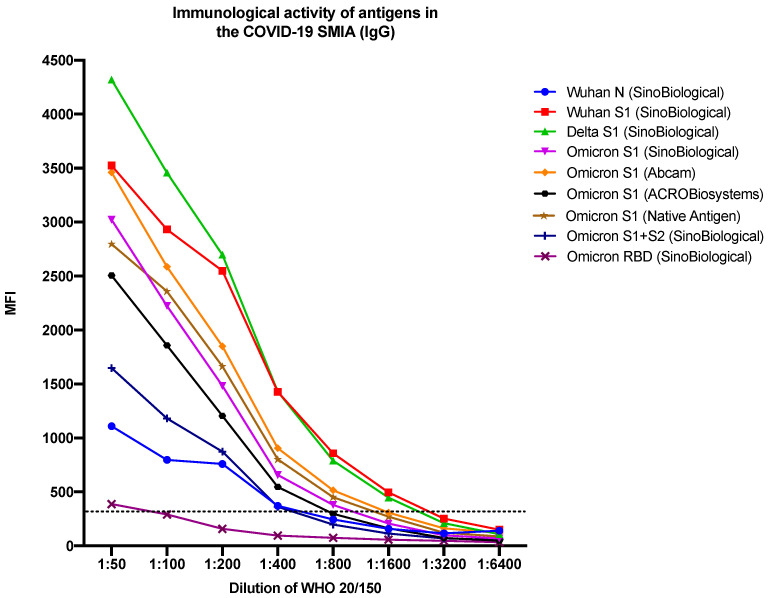
Immunological activity of the sVNT antigens in a COVID-19 SMIA. sVNT—surrogate virus neutralization test; SMIA—suspension multiplex immunoassay; IgG—immunoglobulin G; MFI—median fluorescence intensity; N—nucleocapsid protein; S—spike protein; S1—domain of the spike protein involved in receptor binding; S2—membrane fusion domain of the spike protein; RBD—receptor binding domain; Wuhan, Delta, Omicron—variants of SARS-CoV-2; dotted line indicates the cut-off value. The manufacturers of the antigens are presented within the parenthesis. WHO 20/150—patient reference sample with high levels of antibodies (neutralizing antibodies, anti-RBD IgG, anti-S1 IgG, anti-spike IgG, and anti-N IgG).

Due to the absence of binding between the hACE2 receptor and the B.1.1.529 Omicron S1 antigen, additional Omicron antigens were included—three S1 proteins, one S1 + S2 trimer, and one RBD protein (see Table 1)—as well as the Wuhan N protein, which served as a negative control. The immunological activity of the antigens was evaluated using a COVID-19 suspension multiplex immunoassay (SMIA) based on a previously published method [10]. All antigens proved immunologically active in the COVID-19 SMIA, with the Delta S1 antigen giving the highest signal and the Omicron RBD antigen giving the lowest signal for the WHO SARS-CoV-2 serological reference sample 20/150 with high antibody levels (Figure 4).

**Figure 5:**
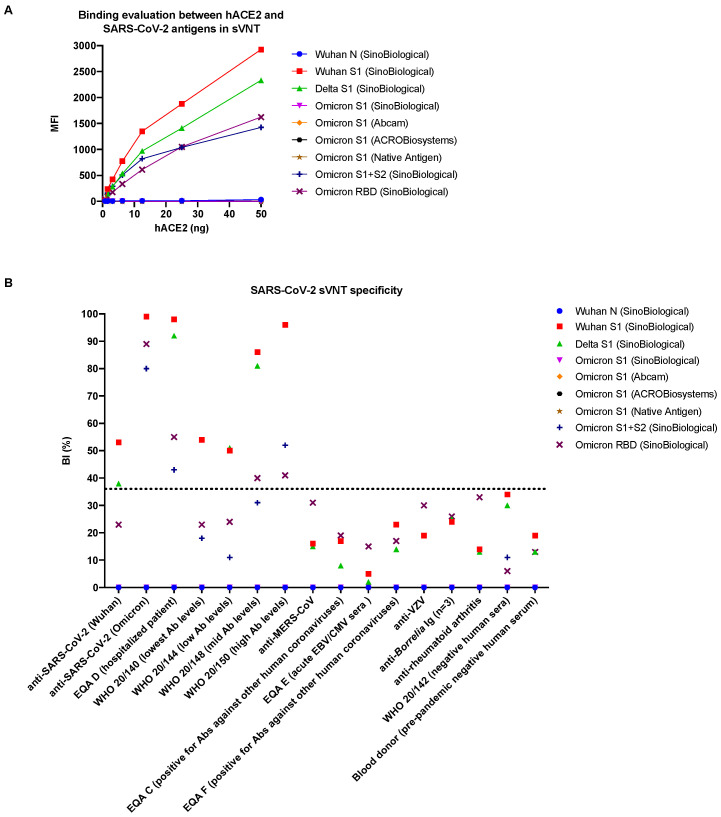
Evaluation of the SARS-CoV-2 sVNT. (**A**) Binding between the hACE2 receptor and SARS-CoV-2 antigens in the SARS-CoV-2 sVNT. Data points represent seven different concentrations of hACE2 (0.78–50 ng, two-fold dilutions). (**B**) Specificity of antigens in the SARS-CoV-2 sVNT. For the anti-SARS-CoV-2 (Omicron), WHO 20/140, WHO 20/150, and anti-Varicella Zoster controls, the BI (%) was identical for the Wuhan S1 and Delta S1 antigens, resulting in overlapping data points in the plot and thereby non-visible data points for the Delta S1 antigen. sVNT—surrogate virus neutralization test; BI (%)—binding inhibition percentage; N—nucleocapsid protein; S—spike protein; S1—domain of the spike protein involved in receptor binding; S2—membrane fusion domain of the spike protein; hACE2—human angiotensin-converting enzyme 2; SARS-CoV-2—Severe acute respiratory syndrome coronavirus 2; Wuhan, Delta, Omicron—variants of SARS-CoV-2; EQA C-F—serum samples that are part of the European Centre for Disease Prevention and Control’s SARS-CoV-2 serological external quality assessment panel; WHO samples—included in the World Health Organization’s international reference panel for anti-SARS-CoV-2; MERS-CoV—Middle East respiratory syndrome coronavirus; EBV—Epstein-Barr virus; CMV—Cytomegalovirus; VZV—Varicella Zoster virus; dotted line indicates the cut-off value. The manufacturers of the antigens are presented within the parenthesis.

The binding between the hACE2 receptor and the included antigens in the SARS-CoV-2 sVNT was evaluated. Specific binding was not observed between the receptor and the Wuhan N protein (negative control) and the tested B.1.1.529 Omicron S1 proteins in the sVNT (Figure 5A). However, binding was observed between the receptor and the S1 + S2 trimer and the RBD of B.1.1.529 Omicron. Additionally, blocking of the binding was observed for the sera obtained from COVID-19 patients infected with the Wuhan and Omicron variants (Figure 5B). The specificity was further evaluated using a control panel, which included sera from individuals known to be positive for antibodies against SARS-CoV-2, MERS-CoV, other human coronaviruses, VZV, and *Borrelia* bacteria as well as sera from patients with rheumatoid arthritis. Negative sera were also included. The specificity was proven to be high for the SARS-CoV-2 sVNT since none of the no-SARS-CoV-2 control samples tested positive for antibodies able to block the binding between the hACE2 receptor and the SARS-CoV-2 antigens (Figure 5B).

### 3.3. Correlation between Wuhan S1 SARS-CoV-2 sVNT (Singleplex Format) and cVNT

The correlation between the Wuhan S1 sVNT and cVNT was evaluated using 54 sera from COVID-19 patients and 18 sera from healthy blood donors. The qualitative results of the SARS-CoV-2 cVNT and the Wuhan S1 SARS-CoV2 sVNT were found to agree 100% for the human samples when using positivity cut-offs of 50% and 80%, respectively, in the cVNT (Table 2).

A 100% correlation was also found when using different international reference panels of control sera and a cut-off of 50% (Table 3).

## 4. Discussion

The presence of NAbs is a good indicator of protective immunity [12]. The current gold standard assay to detect NAbs against SARS-CoV-2 is cVNT, a method requiring infectious virus and a BSL-3 laboratory. In this study, a Luminex-based SARS-CoV-2 sVNT was developed, in which the hACE2 receptor and antibodies compete to bind to the S protein of SARS-CoV-2 conjugated to magnetic beads. The SARS-CoV-2 sVNT proved to be highly specific (Figure 5B) and to have a 100% correlation to a SARS-CoV-2 cVNT regarding qualitative results (Table 2 and Table 3), indicating that the method can be used as a cost-effective, efficient, and rapid substitute for cVNT to predict humoral protection and vaccine efficacy during large-scale serological screenings. Furthermore, the assay is species- and isotype-independent as it does not require species- and isotype-specific conjugates, suggesting that the method can also be a valuable serological tool for the detection of NAbs against SARS-CoV-2 in different animal species. The sVNT can thereby be used in the search for reservoir and intermediate hosts and in the investigation of their importance in the transmission and evolution of SARS-CoV-2.

The Wuhan (Wuhan-Hu-1), Delta (B.1.617.2), and Omicron (B.1.1.529) variants were included to evaluate the ability of the sVNT to detect neutralizing (blocking) antibodies towards three variants of SARS-CoV-2 that have had a dominating role in the transmission during the COVID-19 pandemic. In contrast to the S1 antigens of the Wuhan and Delta variants, the S1 antigen of the B.1.1.529 lineage of the Omicron variant did not bind to the hACE2 receptor in the SARS-CoV-2 sVNT. Conjugation failure of antigens to the beads, i.e., antigens not being coupled to the beads, was excluded as a possible cause of this as we could detect antibodies binding directly to the Omicron S1 proteins coupled to the beads (Figure 4). Binding was, however, observed between the receptor and an S1 + S2 trimer and the RBD of B.1.1.529 Omicron, but in a reduced manner (Figure 5A), suggesting that the S2 domain is needed for the correct presentation of the RBD in Omicron S1 proteins on the beads after conjugation and that mutations in the RBD of the B.1.1.529 Omicron S1 domain may have resulted in reduced/less efficient binding to the hACE2 cell receptor [8,13,14,15]. This contrasts with the studies showing an increased ACE2 binding affinity of the Omicron variant [16,17,18,19].

The control panel of positive sera from COVID-19 patients used in this study was primarily collected in the early phase of the pandemic, i.e., mainly from patients infected with the Wuhan variant. Therefore, the ability of the SARS-CoV-2 sVNT to detect NAbs against new variants of concern needs to be further evaluated. We will therefore continue to update the assay with new antigens and control sera as the pandemic proceeds and new SARS-CoV-2 variants evolve.

## 5. Conclusions

The SARS-CoV-2 sVNT proved to detect immunodominant neutralizing/blocking antibodies targeting different parts of the S protein of the Wuhan, Delta, and Omicron variants. The assay also proved to have a 100% correlation to a cVNT regarding qualitative results, indicating that the sVNT can be used instead of the cVNT for SARS-CoV-2. Binding between the hACE2 receptor and the S1 domain of the B.1.1.529 lineage of Omicron was not observed in the SARS-CoV-2 sVNT, but between the receptor and an S1 + S2 trimer and the RBD in a reduced manner, suggesting reduced receptor binding of the B.1.1.529 lineage of the Omicron variant of SARS-CoV-2. Further investigation regarding receptor binding for the Omicron variant and its lineages, as well as future emerging variants of SARS-CoV-2, are warranted.

Conventional neutralization tests are still the gold standard in most virus systems due to the fact that they measure inhibition of infectivity. However, cVNTs include infectious viruses and are extremely time-consuming and expensive. Surrogate methods are therefore highly appreciated, especially for viruses requiring specialized safety laboratories. Surrogate neutralization tests need to be extensively evaluated in parallel with traditional tests before they can replace them. Our SARS-CoV-2 sVNT shows the most promising results, and we have not so far been able to find any impact of potential limitations of our Luminex-based system. We will continue to compare our results with the cVNT and future variants of SARS-CoV-2.

## Figures and Tables

**Table 1:** Information about the antigens used in the surrogate virus neutralization test for SARS-CoV-2.

Variant (Lineage)	Company	Product No.	Expression System	Purity ^a^	Amino Acids (aa)	No. of aa Changes ^b^	No. of aa Changes in RBD ^c^
Wuhan S1 (Wuhan-Hu-1)	SinoBiological	40591-V08H	HEK293	≥92%	16–685		
Delta S1 (B.1.617.2)	SinoBiological	40591-V08H23	HEK293	92.7%	1–685	8	2 ^e^
Omicron S1 (B.1.1.529)	SinoBiological	40591-V08H41	HEK293	97.0%	1–685	28	15 ^f^
Omicron S1 (B.1.1.529)	Abcam	ab290828	HEK293	>95%	16–671	29	15 ^f^
Omicron S1 (B.1.1.529)	ACROBiosystems	S1N-C52Ha	HEK293	>95%	16–685	28	15 ^f^
Omicron S1 (B.1.1.529)	Native Antigen	REC32006	HEK293	>95%	16–671	29	15 ^f^
Omicron S1 + S2 (B.1.1.529) ^d^	SinoBiological	40589-V08H26	HEK293	94%	16–1234	40	15 ^f^
Omicron RBD (B.1.1.529)	SinoBiological	40592-V08H121	HEK293	97.8%	319–541		15 ^f^
Wuhan N (Wuhan-Hu-1)	SinoBiological	40588-V08B	BVI	96%	1–419		

^a^ Information about the purity was acquired via the lot number, and the purity was determined by sodium dodecyl sulfate polyacrylamide gel electrophoresis (SDS-PAGE); ^b^ relative to Wuhan-Hu-1; ^c^ amino acids: 319-541; ^d^ trimer, extra-cellular domain; ^e^ L452R and T478K; ^f^ G339D, S371L, S373P, S375F, K417N, N440K, G446S, S477N, T478K, E484A, Q493R, G496S, Q498R, N501Y, and Y505H; S—spike protein; S1—domain of the spike protein involved in receptor binding; S2—membrane fusion domain of the spike protein; RBD—receptor binding domain; N—nucleocapsid protein; HEK—human embryonic kidney; BVI—Baculovirus-insect cells.

**Table 2:** Correlation between conventional- and surrogate virus neutralization test for SARS-CoV-2, using human sera.

		cVNT (Cut-Off: 50%)		cVNT (Cut-Off: 80%)	
COVID-19 patients (*n* = 54)		Pos	Neg	Pos	Neg
sVNT (Cut-off: 36%)	Pos	54	0	54	0
	Neg	0	0	0	0
Blood donors (*n* = 18)		Pos	Neg	Pos	Neg
sVNT (Cut-off: 36%)	Pos	0	0	0	0
	Neg	0	18	0	18

Pos—positive; neg—negative; sVNT—surrogate virus neutralization test; cVNT—conventional virus neutralization test. 80%/50%/36%—positivity cut-off. Sample dilution: 1:20.

**Table 3:** Comparison of conventional and surrogate SARS-CoV-2 VNT (the latter in singleplex format and with Wuhan S1 as antigen), using reference and standard sera provided by the European Centre for Disease Prevention and Control (ECDC) and the World Health Organization (WHO).

		cVNT	sVNT
ID	Control Serum/Plasma	Inhibition (%)	Inhibition (%)
		(1:20)	(1:20)
EQA A	SARS-CoV-2 Abs (Hospitalized patient)	100 *	91
EQA B	SARS-CoV-2 Abs (Mild infection)	100	94
EQA C	Negative pre-pandemic sera (Other human CoV)	0	0
EQA D	SARS-CoV-2 Abs (Hospitalized patient)	100	97
EQA E	Acute EBV/CMV sera (Other human CoV)	0	0
EQA F	Negative pre-pandemic sera (Other human CoV)	0	0
EQA G	SARS-CoV-2 Abs (Mild infection)	100	80
EQA H	SARS-CoV-2 Abs (Hospitalized patient)	100	89
JCR 017	SARS-CoV-2 Abs (Standard)	98	80
JCR 018	SARS-CoV-2 Abs (Standard)	92	87
20/136	SARS-CoV-2 Abs (Standard)	100	96
20/140 (20/268)	SARS-CoV-2 Abs + NAbs (Lowest Ab levels)	53	54
20/142 (20/268)	Negative human sera	6	34
20/144 (20/268)	SARS-CoV-2 Abs + NAbs (Low Ab levels)	92	50
20/148 (20/268)	SARS-CoV-2 Abs + NAbs (Mid Ab levels)	97	86
20/150 (20/268)	SARS-CoV-2 Abs + NAbs (High Ab levels)	100	96

EQA A–H—ECDC’s SARS-CoV-2 serological external quality assessment panel; JCR 017 and 018—ECDC’s SARS-CoV-2 serological standards; * 1:100; Abs—antibodies; NAbs—neutralizing antibodies; SARS-CoV-2—Severe acute respiratory syndrome coronavirus 2; CoV—coronavirus; EBV—Epstein-Barr virus; CMV—Cytomegalovirus; grey—above positivity cut-off (50% (cVNT), 36% (sVNT)).

## Data Availability

Data is available upon request.
